# Treatment-Resistant Very Late Onset Schizophrenia-Like Psychosis: A Case Study

**DOI:** 10.1192/bjo.2026.11767

**Published:** 2026-06-30

**Authors:** Ryan Bellman, Carolina Pressanto, Anu Ipe

**Affiliations:** 1 East Kent Hospitals University NHS Foundation Trust, Canterbury, United Kingdom; 2 Kent and Medway Mental Health NHS Trust, Maidstone, United Kingdom

## Abstract

**Aims:**

A 77-year-old male with no prior contact with psychiatric services, and a medical history of a myocardial infarction and osteoarthritis, presented in 2022 with a 10-week history of psychotic symptoms. These included auditory hallucinations as well as persecutory and somatic delusions. He was diagnosed with Very Late Onset Schizophrenia-Like Psychosis (VLOSLP) and detained under the Mental Health Act due to non-adherence and risk.

**Methods:**

Since 2022, several antipsychotics (olanzapine, aripiprazole oral and depot, risperidone) have been trailed, however symptoms persisted with repeated relapses linked to non-adherence. Zuclopenthixol decanoate led to partial stabilisation but with residual psychosis. In June 2023, he attempted suicide by hanging, requiring admission to Intensive Care Unit. Antidepressants (sertraline and then later mirtazapine/vortioxetine) were added for comorbid depressive symptoms.

From September 2023, clozapine was initiated due to treatment-resistance. Titration was limited by hypotension, sedation, constipation, and poor engagement, compounded by persistent psychotic symptoms and delusional beliefs about blood loss and poisoning. Magnetic Resonance Imaging of the brain and autoimmune encephalitis screening were normal.

Between 2024 and 2025, recurrent crises occurred due to non-adherence and entrenched delusions. Clozapine was intermittently stopped and restarted with cardiology input following concerns about heart failure. The multidisciplinary team decided on slow clozapine titration, with use of intramuscular clozapine when non-adherent, and to continue this long-term.

**Results:**

VLOSLP is defined by a patient’s first episode of psychosis after 60 years of age. VLOSLP poses significant diagnostic challenges as up to 60% of cases are secondary to organic causes, and there are significant overlapping features between VLOSLP and neurodegenerative disorders, although the pathophysiology still remains unclear. After 60, the incidence of schizophrenia increases around 11% every 5 years. Clinically, VLOSLP has a lower prominence of negative symptoms and formal thought disorders, and a higher prominence of partition and persecutory delusions, multimodal hallucinations and cognitive impairment. Management requires cautious use of antipsychotics at lower doses due to increased morbidity and mortality risks associated with elderly patients. The literature shows treatment-resistant VLOSLP may benefit from options such as clozapine, electroconvulsive therapy, or neuromodulation strategies.

**Conclusion:**

Currently, the patient remains under inpatient management on clozapine, targeting a serum clozapine level of 0.35-0.45mg/L, with ongoing monitoring for side effects. He still experiences fixed delusions and auditory hallucinations, requiring ongoing encouragement for adherence, with occasional use of intramuscular clozapine. Overall, there is limited evidence on VLOSLP and treatment-resistant VLOSLP, and further research is required for optimal diagnostic and treatment pathways.

